# Resveratrol Suppresses Matrix Metalloproteinase-2 Activation Induced by Lipopolysaccharide in Mouse Osteoblasts via Interactions with AMP-Activated Protein Kinase and Suppressor of Cytokine Signaling 1

**DOI:** 10.3390/molecules23092327

**Published:** 2018-09-12

**Authors:** Yaqiong Yu, Xiaolin Li, Jing Mi, Liu Qu, Di Yang, Jiajie Guo, Lihong Qiu

**Affiliations:** 1 Department of Endodontics, School of Stomatology, China Medical University, Shenyang 110002, China; yuyaqiong@cmu.edu.cn (Y.Y.); aboutlxl@sina.com (X.L.); mijing1993@sina.com (J.M.); liu.qu@foxmail.com (L.Q.); missyangdi@hotmail.com (D.Y.); jiajieguo@hotmail.com (J.G.); 2 Liaoning Province Key Laboratory of Oral Diseases, Shenyang 110002, China

**Keywords:** cell signaling, resveratrol, lipopolysaccharide, apical periodontitis

## Abstract

*Porphyromonas endodontalis* (*P. endodontalis*) lipopolysaccharide (LPS) is associated with the progression of bone resorption in periodontal and periapical diseases. Matrix metalloproteinase-2 (MMP-2) expression and activity are elevated in apical periodontitis and have been suggested to participate in bone resorption. Therefore, inhibiting MMP-2 activation may be considered a therapeutic strategy for treating apical periodontitis. Resveratrol is a natural non-flavonoid polyphenol that has been reported to have antioxidant, anti-cancer, and anti-inflammatory properties. However, the capacity of resveratrol to protect osteoblast cells from *P. endodontalis* LPS insults and the mechanism of its inhibitory effects on MMP-2 activation is poorly understood. Here, we demonstrate that cell viability is unchanged when 10 mg L^−1^ *P. endodontalis* LPS is used, and MMP-2 expression is drastically induced by *P. endodontalis* LPS in a concentration- and time-dependent manner. Twenty micromolar resveratrol did not reduce MC3T3-E1 cell viability. Resveratrol increased AMP-activated protein kinase (AMPK) phosphorylation, and Compound **C**, a specific AMPK inhibitor, partially abolished the resveratrol-mediated phosphorylation of AMPK. In addition, AMPK inhibition blocked the effects of resveratrol on MMP-2 expression and activity in LPS-induced MC3T3-E1 cells. Treatment with resveratrol also induced suppressor of cytokine signaling 1 (SOCS1) expression in MC3T3-E1 cells. SOCS1 siRNA negated the inhibitory effects of resveratrol on LPS-induced MMP-2 production. Additionally, resveratrol-induced SOCS1 upregulation was reduced by treatment with compound **C**. These results demonstrate that AMPK and SOCS1 activation are important signaling events during resveratrol-mediated inhibition of MMP-2 production in response to LPS in MC3T3-E1 cells, and there is crosstalk between AMPK and SOCS1 signaling.

## 1. Introduction

Apical periodontitis is an inflammatory lesion in periodontal tissues that is caused mostly by bacterial elements derived from an infected root canal system. The association between apical periodontitis and cardiovascular diseases has been reported [1]. *Porphyromonas endodontalis* (*P. endodontalis*) is a black pigmented Gram-negative anaerobic microorganism, that is a key pathogen in endodontal infections and apical periodontitis, which is attributed to its high prevalence and pathogenic factors [2,3,4]. Lipopolysaccharide (LPS) produced by *P. endodontalis* triggers the production of a variety of cytokines that participate in bone destruction in the course of apical periodontitis [5,6,7]. Matrix metalloproteinase-2 (MMP-2) is a member of MMP family of zinc-dependent endopeptidases that have consistently been shown to be associated with the degradation of the extracellular connective matrix (ECM) and may also be involved in bone destruction [8,9]. Many studies have demonstrated that MMP-2 expression and activity are elevated in apical periodontitis and have been suggested to play a key role in the progression of this disease [10,11,12]. Furthermore, one study showed that there is a possible relationship between MMP-2 and tooth pain, which is related to maintenance of the neuropathic pain process via cleavage of interleukin-1 β and astrocyte activation [11]. Therefore, abnormally elevated MMP-2 activation must be tightly regulated or properly controlled.

Resveratrol (3,5,4′-trihydroxy-*trans*-stilbene) is a polyphenol commonly found in grapes [13,14,15], red wine [16], mulberries and other plants, and its wide range of pharmacological properties, including cardioprotective, antioxidant, neuroprotective and anticancer effects, is well-characterized [17,18]. Some studies have also reported a powerful effect of resveratrol in delaying the progress of ligature placement-associated alveolar bone resorption during periodontitis [19,20,21]. AMP-activated protein kinase (AMPK) is a serine/threonine kinase that can sense cellular changes in energy and regulate energy metabolic homeostasis. AMPK is a heterotrimer complex comprising a catalytic α subunit and regulatory β and γ subunits. Phosphorylation of threonine 172 in the catalytic domain of the α subunit by an upstream AMPK kinase is essential for its activation [22]. Previous studies have shown that AMPK might also act as a novel suppressor of inflammation [17,19].

The suppressor of cytokine signaling (SOCS) proteins is a family of intracellular proteins, including at least eight SOCS members: SOCS1-7 and cytokine-inducible SH2 protein (CIS) [23]. All SOCS proteins share a central SRC-homology 2 (SH2) domain, an amino-terminal domain of variable length and sequence, and a conserved SOCS box at the C-terminus [23]. Specifically, SOCS1 and SOCS3 take part in a negative feedback loop to attenuate inflammatory responses in osteoblasts [24,25]. Current studies demonstrated that resveratrol is able to alleviate the inflammatory responses of LPS-stimulated macrophages and microglial cells via SOCS1, as a negative regulator of cytokine signaling [26,27]. However, the mechanism of resveratrol’s beneficial anti-inflammatory effects in osteoblast are poorly understood.

In the present study, we demonstrate that resveratrol attenuates the expression of MMP-2 induced by *P. endodontalis* LPS in mouse osteoblasts. Resveratrol treatment also results in AMPK activation and upregulation of SOCS1, leading to anti-inflammatory effects.

## 2. Results

### 2.1. MMP-2 Is Elevated in MC3T3-E1 Cells by P. endodontalis LPS Treatment

**Figure 1:**
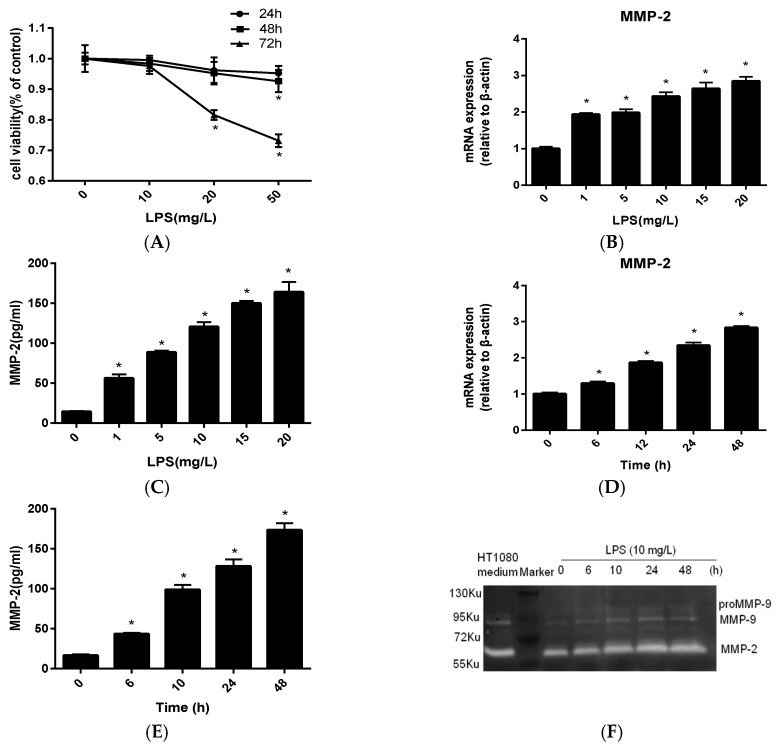
MMP-2 is elevated by *P. endodontalis* lipopolysaccharide (LPS) treatment in MC3T3-E1 cells. (**A**) MC3T3-E1 cells were treated with *P. endodontalis* LPS at the indicated concentrations. Cell viability was evaluated by MTT assays after 24 h, 48 h and 72 h of treatment. MC3T3-E1 cells were treated with the indicated concentrations *P. endodontalis* LPS for 24 h, and MMP-2 expression was determined by real-time PCR (**B**) and ELISA (**C**). MC3T3-E1 cells were exposed to 10 mg L^−1^ *P. endodontalis* LPS for the indicated time. MMP-2 expression was determined by real-time PCR (**D**) and ELISA (**E**). (**F**) Gelatinolytic activity produced by MMP-2 was detected by gelatin zymography in MC3T3-E1 cells treated with 10 mg L^−1^ *P. endodontalis* LPS for the indicated time. HT1080 medium indicates conditioned medium collected from HT1080 (human fibrosarcoma) cells that was used as a positive control. U is the atomic mass unit. 1 Ku is approximately equal to 1 KDa. * *p* < 0.05, as compared to the control group.

After stimulation with different amounts of *P. endodontalis* LPS (0, 10, 20, 50 mg L^−1^), the viability of mouse osteoblastic MC3T3-E1 cells was assessed by MTT assays at 24 h, 48 h and 72 h. Cell viability decreased in a concentration-dependent manner in response to *P. endodontalis* LPS, to 92.6% with 50 mg/L *P. endodontalis* LPS at 48 h and 81.6% with 20 mg L^−1^ *P. endodontalis* LPS, to 73.1% using 50 mg/L *P. endodontalis* LPS at 72 h (Figure 1A). To elucidate MMP-2 mRNA and protein expression, MC3T3-E1 cells were treated with 0, 1, 5, 10, 15, 20 mg L^−1^ *P. endodontalis* LPS for 24 h. MMP-2 mRNA and protein expression increased in response to increasing *P. endodontalis* LPS concentrations (Figure 1B,C). Within 48 h of observation, MMP-2 mRNA and protein expression gradually increased with extending stimulus periods with 10 mg L^−1^ *P. endodontalis* LPS (Figure 1D,E). Gelatin zymography was performed to determine whether *P. endodontalis* LPS treatment affects MMP-2 activity. Gelatinases separated by molecular weight were visualized as transparent spots on Coomassie Blue-stained gels. Conditioned medium from HT1080 cells (human fibrosarcoma cells) was used as a positive control. The intensity of MMP-2 spots increased with the prolonged stimulated time.

### 2.2. Resveratrol Inhibit P. endodontalis LPS-Stimulated MMP-2 Expression by Activating AMPK in MC3T3-E1 Cells

**Figure 2:**
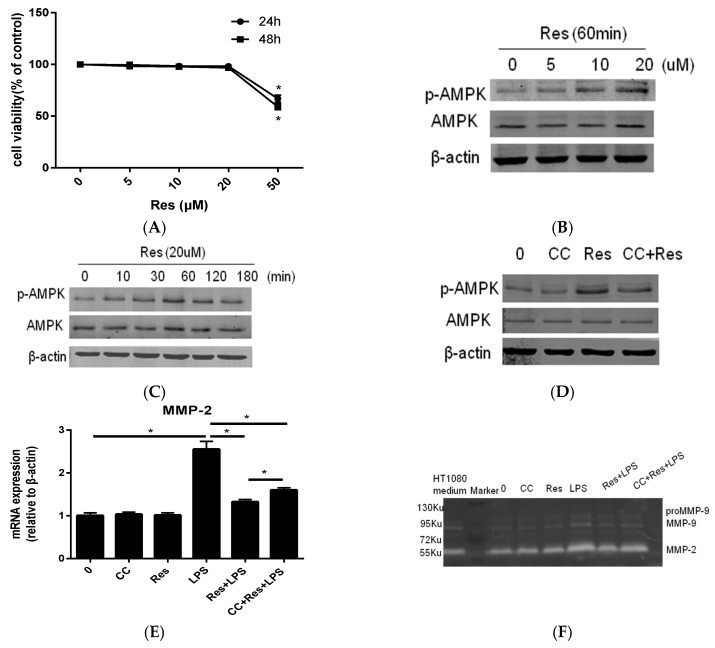
The involvement of AMPK in the inhibitory effects of resveratrol on MMP-2 expression in MC3T3-E1 cells stimulated by *P. endodontalis* LPS. (**A**) MC3T3-E1 cells were incubated with 0, 5, 10, 20, 50 µM resveratrol (Res) for 24 and 48 h, after which MC3T3-E1 cells viability was determined by MTT analysis. MC3T3-E1 cells were incubated with various concentrations resveratrol for 60 min (**B**); MC3T3-E1 cells were treated with 20 μM resveratrol for the indicated time (**C**); MC3T3-E1 cells were pretreated with 20 μM resveratrol for 1 h, or not, in the absence or presence of 10 μM Compound **C** (CC) for 30 min (**D**). Treated MC3T3-E1 cell lysates were subjected to western blot analysis using antibodies against total AMPK and AMPK phosphorylated at Thr172 on the α-subunit (shown as p-AMPK). MC3T3-E1 cells were pretreated with 20 μM resveratrol for 1 h, or not, in the absence or presence of 10 μM Compound **C** for 30 min and then exposed to 10 mg L^−1^ *P. endodontalis* LPS for 24 h. MMP-2 mRNA expression was detected by real-time PCR (**E**) and the cell culture medium was then harvested to determine the collagenase activity of MMP-2 by gelatin zymography (**F**). HT1080 medium indicates conditioned medium collected from HT1080 cells (human fibrosarcoma cells) that served as a positive control. U is the atomic mass unit. 1 Ku is approximately equal to 1 KDa. * *p* < 0.05, as compared to the control group.

Initially, the toxicities of various does of resveratrol (0, 5, 10, 20, 50 μM) on MC3T3-E1 cells was measured by MTT assay at 24 h and 48 h. Concentrations below 20 μM resveratrol showed no toxicity as compared to the control group, while 50 μM resveratrol caused progressive osteoblast cell death from 24 to 48 h (Figure 2A). We thus used 20 μM resveratrol for the subsequent experimentation. To further determine whether AMPK phosphorylation is induced by resveratrol, antibodies against phospho-AMPKα (Thr172) and AMPKα were used in resveratrol-treated cells. Treatment of cells with resveratrol increased AMPKα phosphorylation at Thr172 in a concentration- and time-dependent manner (Figure 2B,C). Compound **C**, an AMPK inhibitor, effectively antagonized AMPK phosphorylation that was caused by resveratrol administration (Figure 2D). The inhibitory effects of resveratrol on *P. endodontalis* LPS-induced MMP-2 mRNA expression and collagenase activity were attenuated in MC3T3-E1 cells, as well (Figure 2E,F). These results indicate that AMPKα is involved in resveratrol-mediated suppression of MMP-2 mRNA expression in osteoblasts stimulated by *P. endodontalis* LPS.

### 2.3. SOCS1 Was Involved in the Inhibitory Effects of Resveratrol on MMP-2 Expression in MC3T3-E1 Cells Stimualted by P. endodontalis LPS

**Figure 3:**
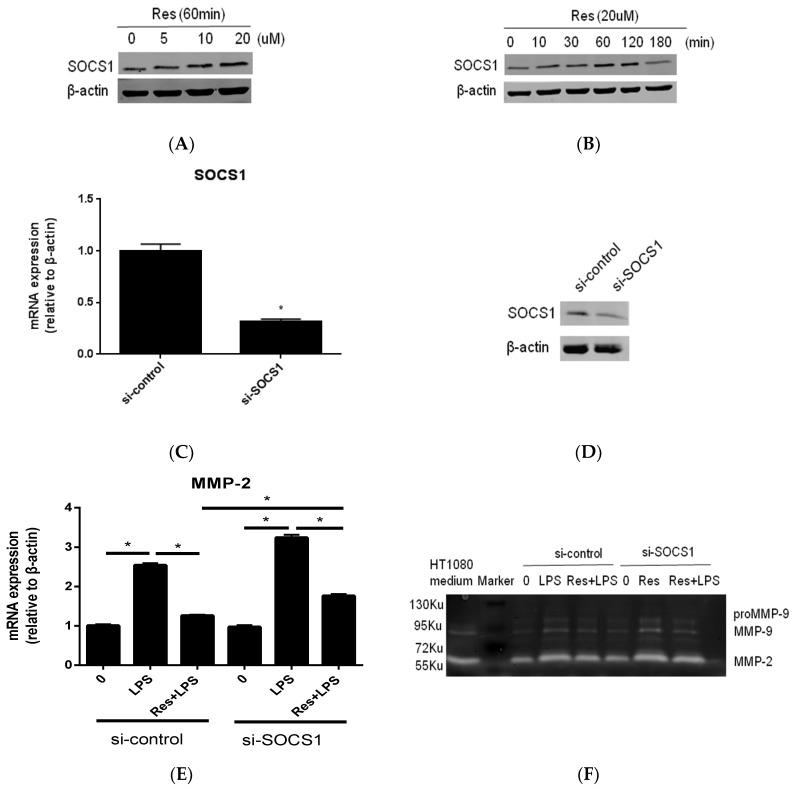

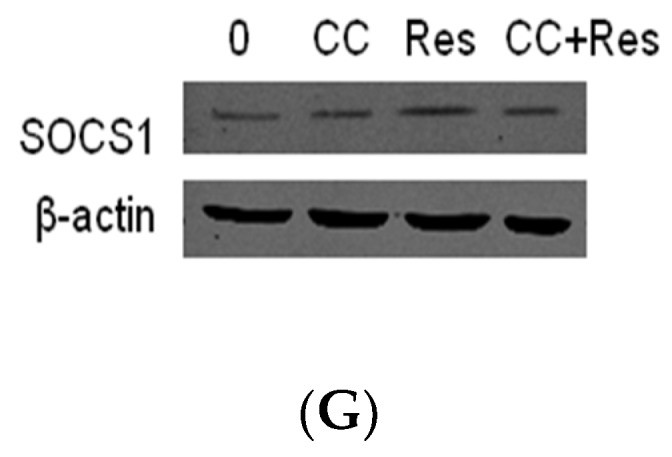
SOCS1 is required for resveratrol-induced suppression of MMP-2 expression induced by *P. endodontalis* LPS in MC3T3-E1 cells. MC3T3-E1 cells were incubated with various concentrations resveratrol (Res) for 60 min (**A**). MC3T3-E1 cells were treated with 20 μM resveratrol for the indicated time periods (**B**). SOCS1 protein levels were detected by western blot analysis. MC3T3-E1 cells were transfected with si-control or si-SOCS1. SOCS1 expression was detected by real-time PCR (**C**) and western blot (**D**). MC3T3-E1 cells were transfected with si-control or si-SOCS1. Transfected cells were stimulated with 10 mg L^−1^ *P. endodontalis* LPS for 24 h, or not, in the absence or presence of 20 μM resveratrol for 1 h, and then MMP-2 mRNA expression and collagenase activity were detected by real-time PCR (**E**) and gelatin zymography (**F**), respectively. HT1080 medium indicates conditioned medium collected from HT1080 (human fibrosarcoma) cells that was used as a positive control. U is the atomic mass unit. 1 Ku is approximately equal to 1 KDa. (**G**) MC3T3-E1 cells were pretreated with 20 μM resveratrol for 1 h, or not, in the absence or presence of 10 μM Compound **C** (CC) for 30 min. SOCS-1 protein levels were detected by western blot analysis. * *p* < 0.05 as compared to the control group.

We next examined whether SOCS1 induction is involved in the effects of resveratrol. Stimulating cells with various concentrations of resveratrol increased SOCS1 protein (Figure 3A). SOCS1 protein expression increased, when osteoblasts were exposed to 20 μM resveratrol for 60 min (Figure 3B). We then used siRNA against SOCS1 to examine whether SOCS1 expression is involved in exerting the effects of resveratrol. SOCS1 mRNA expression was significantly down-regulated following transfection with siSOCS1 (Figure 3C). Western blot analysis was then performed to identify SOCS1 protein expression after siRNA transfection (Figure 3D). Transfection with SOCS1 siRNA increased MMP-2 mRNA expression and collagenase activity in LPS-stimulated MC3T3-E1 cells and negated the inhibitory effects of resveratrol on LPS-induced MMP-2 mRNA expression and collagenase activity (Figure 3E,F). Furthermore, resveratrol-induced SOCS1 expression was reduced by treatment with Compound **C** (Figure 3G). These results indicate that SOCS1 is required for the inhibitory effects of resveratrol on MMP-2 mRNA expression in *P. endodontalis* LPS treated osteoblast cells, and that AMPKα activation is involved in SOCS1 upregulation by resveratrol.

## 3. Discussion

Persistent periapical inflammation often leads to destruction of periapical tissue and even alveolar bone resorption. Pathologic changes of the periapical tissues in apical periodontitis are caused by microbes, their toxins and metabolic byproducts. *P. endodontalis*, a Gram-negative anaerobic bacteria, is frequently encountered in apical periodontitis with a prevalence of nearly 65% [2]. Our previous studies demonstrated that the release of a series of pro-inflammatory cytokines induced by *P. endodontalis* LPS may be related to bone destruction in apical periodontitis [5,6,7]. MMP-2 can cleave type IV collagen, specifically, which is a major structural component of the ECM. As periapical tissue destruction is usually accompanied by ECM degradation, MMP-2 is considered responsible for periapical damage during inflammation [10,11,12]. In the bone resorption microenvironment, two gelatinases are increased by stimulating factors and are involved in bone destruction, one of which is MMP-2 produced mainly by osteoblasts, and the other one is MMP-9 produced by both osteoblasts and osteoclasts [28]. Therefore, we herein investigated the induction of MMP-2 by *P. endodontalis* LPS and the mechanism by which resveratrol reduces MMP-2 production in osteoblasts. Osteoblasts express many matrix metalloproteinases (MMPs) in response to inflammatory processes including MMP-2, MMP-3, MMP-8, MMP-9 and MMP-13 [8,9]. Excessive gelatinase activation is a leading cause of detrimental outcomes in apical periodontitis. Given the role of MMP-2 in the degradation of fibrillar collagen, which is one of the main constituents of the periodontium and alveolar bone, it is well-understood that MMP-2 may be used as a biomolecule for the healing of apical lesions [29]. Most studies support the hypothesis that LPS from *Porphyromonas gingivalis*, which is another common Gram-negative bacteria associated with periapical infection, induces MMP-2 production in different cell types [30,31]. Here, we found that 10 mg L^−1^ *P. endodontalis* LPS did not affect osteoblast viability (Figure 1A); however, it clearly induces the expression and gelatinolytic activities of MMP-2 (Figure 1D–F). Therefore, it is important to further interrogate strategies for diminishing active MMP-2 levels.

Resveratrol is a stilbenoid compound produced by several plants, including grapes and some traditional Chinese medicinal plants, such as *Polygonum Cuspidatum* [32], and it has been reported to exhibit a variety of biological activities [17,18]. Emerging evidence implicates AMPK as a metabolic sensor and regulator of cellular energy homeostasis mediating the anti-inflammatory effects of resveratrol in neurodegenerative disease [17] and endothelial dysfunction [33]. It has been reported that resveratrol-mediated AMPK activation is not ubiquitously observed in different cell types, for example, resveratrol fails to activate AMPK even at high concentrations in preadipocytes [34]. In the present study, we show that resveratrol promote AMPK phosphorylation in MC3T3E1 cells under basal conditions (Figure 2B,C). Several studies have reported that metformin, an activator of AMPK, regulates endothelial cell and fibroblast migration by inhibiting MMP-2 expression [35,36]. However, only a few studies have been conducted exploring MMP-2 inhibition by resveratrol via an AMPK-dependent mechanism. To further confirm the role of AMPK in mediating the anti-inflammatory effect of resveratrol, we used the AMPK inhibitor Compound **C**, and finding that Compound **C** abrogates AMPK phosphorylation (Figure 2D). Indeed, the inhibitor also blocks resveratrol-mediated anti-inflammatory effects in human embryonic stem cells [17]. Similar phenomena were observed in rat periodontitis model, which demonstrate that AMPK activation by resveratrol prevents alveolar bone resorption and periodontal inflammation induced by ligature placement [19]. This suggests that AMPK is widely involved in the anti-inflammatory effect of resveratrol.

Increasing evidence has shown that SOCS1 negatively regulates inflammation by taking part in a negative feedback loop to attenuate cytokine signaling [37]. SOCS1-defcient macrophages secrete more pro-inflammatory cytokines than wild-type cells, including tumor necrosis factor-α and interleukin-6 [38]. It also has been reported that SOCS1 is a negative modulator of the inflammatory response in apical periodontitis [39]. Importantly, recent studies have reported that resveratrol is a very potent inducer of SOCS1 protein expression in microglia, head and neck tumor cells, and macrophages [26,27,40]. In present study, we observed that SOCS1 protein is considerably expressed, even under the sedentary conditions, in MC3T3E1 cells (Figure 3A,B). Our findings are consistent with those of several prior reports [26,27], and we hypothesize that basal expression of SOCS1 protein is likely stored for rapid responses to contrast inflammation or cellular stress. SOCS1 has been reported to inhibit signal transducers and activators of transcription (STAT) phosphorylation by inhibiting the activation of a family of receptor-bound tyrosine kinases known as the Janus kinases (JAKs) [23]. Interestingly, MMP-2 is a STAT-regulated gene product [41]. It has also been reported that SOCS1 overexpression in human epithelial lung carcinoma cells significantly inhibits MMP-2 levels [42], which is consistent with our results. We found that compound **C** reduces the positive effect of resveratrol on SOCS1 expression in osteoblasts (Figure 3G). As far as we know, this is the first report to demonstrate that the inter-regulation between AMPK and SOCS1 mediates the anti-inflammatory effects of resveratrol. One prior report demonstrated that AMPK may interact with SOCS1 by inducing JAK2 degradation in anemia-induced inflammation [43].

Our previous studies indicated that resveratrol is a potent agonist for SIRT1, and SIRT1 upregulation effectively exerts anti-inflammation molecule expression in osteoblasts [7]. Resveratrol has previously been reported to regulate SIRT1 by activating AMPK, as AMPK inhibitory activity remarkably abolished resveratrol-induced SIRT1 expression [44]. Interestingly, SIRT1 activation also plays a key role in regulating SOCS1 expression in microglia [45]. Further work is needed to clearly investigate whether SIRT1 acts as a modulator of the AMPK/SOCS1 pathway-mediated anti-inflammatory effects of resveratrol in osteoblasts.

Altogether, our results indicate that resveratrol suppresses MMP-2 expression induced by *P. endodontalis* LPS by regulating the AMPK/SOCS1 pathway in osteoblasts. These findings provide further pharmacological evidence for the beneficial effects of resveratrol on local bone resorption during apical periodontitis.

## 4. Materials and Methods

### 4.1. Cell Culture

Murine osteoblastic MC3T3-E1 cells were acquired from the Cell Bank of the Chinese Academy of Sciences (Shanghai, China) and were cultured in α-MEM (Invitrogen, Carlsbad, CA, USA) with 10% fetal bovine serum (Invitrogen) at 37 °C in a humidified incubator with an atmosphere of 5% CO_2_ and 95% air.

### 4.2. Bacterial Culture and LPS Extraction

*P. endodontalis* (ATCC35406) was acquired from the Central Laboratory of Capital Medical University (Beijing, China) and cultured anaerobically at 37 °C. LPS was extracted from bacterial cell walls using an established hot phenol-water method, as previously described [5,6,46].

### 4.3. MTT Assay

The day before treatment, MC3T3-E1 cells were seeded into 96-well plates and treated with 0, 5, 10, 20 or 50 μM resveratrol (Sigma-Aldrich, St. Louis, MO, USA) for 24 and 48 h; or with 0, 10, 20 or 50 mg/L *P. endodontalis* LPS for 24 h, 48 h and 72 h. Mitochondrial activity was assayed with an MTT (3-[4-dimethylthiazol-2-yl]-2,5-diphenyltetrazolium bromide) (Sigma-Aldrich) assay. Absorbance at 570 nm was determined in solubilized cells using an Infinite M200 Multimode Reader (Tecan, Männedorf, Switzerland). The cell growth rate was expressed as a percentage of the control.

### 4.4. ELISA Analysis

The day before treatment, MC3T3-E1 cells were seeded into 35-mm dishes and treated with different doses of *P. endodontalis* LPS for the indicated time periods. MMP-2 concentrations were measured in triplicate using supernatants obtained from cultures using ELISA (Boster Company, Wuhan, China) according to the manufacturer’s instructions. Absorbance at 450 nm was determined in cellular supernatants using an Infine M200 Multimode Reader (Tecan). The concentration of proteins were calculated in triplicate using the standard protein curve.

### 4.5. Gelatin Zymography

Grouping situation was detailed in the Section 2. The gelatinolytic activities of MC3T3-E1 cell supernatants were determined via gelatin zymography assay (Xinfan Technology, Shanghai, China) according to the manufacturer’s instructions. HT1080 (human fibrosarcoma) cell media conditioned for 24 h was used as a positive control, as it is enriched with gelatinases [47]. Supernatants were collected from culcured cells and examined with 8% polyactylamide gels containing 0.1% gelatin. Gels were washed with 2.5% Triton X-100 buffer three times following electrophoresis. Gels were incubated in reaction buffer (50 mM Tris HCL (pH 7.6), 5 mM CaCl_2_ and so on) at 37 °C overnight. Gels were then stained with Coomassie Blue R-250 (Sigma-Aldrich) and destained appropriately. The gels image was obtained by Odyssey CLx imaging system (LI-COR, Lincoln, NE, USA).

### 4.6. Transfection

MC3T3-E1 cells were transfected with mouse SOCS1 siRNA (RiboBio Company, Guangzhou, China) using Lipofectamine^TM^ Reagent (Invitrogen) at 70–80% confluence, according to the manufacturer’s protocol. SOCS-1 suppression was determined by real-time PCR and western blotting after transfection for 48 h. Cells were stimulated 48 h post-transfection with 10 mg L^−1^ *P. endodontalis* LPS for 24 h, or not, in the absence or presence of 20 μM resveratrol for 1 h.

### 4.7. RNA Preparation and Real-Time PCR

Total RNA was extracted from MC3T3-E1 cells using RNAiso Plus reagent (TaKaRa, Kyoto, Japan), followed by phenol extraction and ethanol precipitation. RNA was then reversed transcribed into cDNA using PrimeScript^TM^ RT Master Mix (TaKaRa). Real-time PCR was performed using SYBR^®^ Premix Ex Taq^TM^ II (TaKaRa). Amplified reactions were quantified on an ABI 7500 Real-time PCR system (Applied Biosystems, Foster City, CA, USA).

Primers used for the desired sequence are as shown: mouse MMP-2 (forward) 5′-ACCCA GATGTGGCCAACTAC-3′; mouse MMP-2 (reverse) 5′-TACTTTTAAGGCCCGAGCAA-3′; mouse SOCS1 (forward) 5′-CACCTTCTTGGTGCGCG-3′; mouse SOCS1 (reverse) 5′-AAGCCATCTTCA CGCTGAGC-3′; mouse β-actin (forward) 5′-CAATAGTGATGACCTGGCCGT-3′; mouse β-actin (reverse) 5′-AGAGGGAAATCGTGCGTGAC-3′. Relative mRNA expression levels were quantified, as compared to β-actin using the 2^−ΔΔ*C*t^ method.

### 4.8. Western Blotting

Following appropriate treatment, MC3T3-E1 cells were lysed using a protein extraction kit (Bio-Rad, Hercules, CA, USA). Equal quantities of protein samples were separated by SDS-PAGE and transferred onto polyvinylidene difluoride membranes (Millipore, Bedford, MA, USA). Membranes were blocked in 5% fat-free milk for 2 h at room temperature before being probed with anti-AMPKα antibody (Cell Signaling Technology, Inc., Danvers, MA, USA) (diluted at 1:1000 *v*:*v*), anti-p-AMPKα (Thr172) antibody (Cell Signaling Technology) (diluted at 1:1000 *v*:*v*), anti-SOCS1 antibody (Abcam, Cambridge, MA, USA) (diluted at 1:1000 *v*:*v*), or anti-β-actin antibody (Proteintech, Rosemont, IL, USA) (diluted at 1:2000 *v*:*v*) overnight at 4 °C. Protein bands were detected using an Odyssey CLx imaging system (LI-COR) followed by incubation for 2 h at ambient temperature with goat anti rabbit secondary antibody conjugated with DyLight fluorescent 800 (LI-COR) (diluted at 1:20,000 *v*:*v*).

### 4.9. Statistical Analysis

According to Shapiro-Wilk tests, all data were normally distributed. Homogeneity of variances was tested with a Levene’s test. Results are expressed as the mean ± standard deviation (SD). The significance of differences between treated groups and the control group were analyzed by one-way ANOVA followed by Dunnett’s *t*-test. Statistical comparisons between two groups with different treatment factors were performed using one-way ANOVA followed by Bonferroni post hoc analysis. Differences were considered significant when *p* < 0.05. Graphs were constructed using GraphPad Prism 5.0 software (GraphPad Software, Inc., La Jolla, CA, USA).
